# P17 Experience of longer-term use of dalbavancin in the OPAT setting

**DOI:** 10.1093/jacamr/dlaf239.021

**Published:** 2026-01-05

**Authors:** Joseph Suich, Joanne Delahay, Sally O’Neill, Hayleigh Cutler, Stephanie Fussey, Cameron Bowen, Chloe Walsh

**Affiliations:** Hull Teaching Hospitals NHS Trust, Hull, UK; Hull Teaching Hospitals NHS Trust, Hull, UK; Hull Teaching Hospitals NHS Trust, Hull, UK; Hull Teaching Hospitals NHS Trust, Hull, UK; Hull Teaching Hospitals NHS Trust, Hull, UK; Hull Teaching Hospitals NHS Trust, Hull, UK; Hull Teaching Hospitals NHS Trust, Hull, UK

## Abstract

**Background:**

Dalbavancin represents a novel, second generation lipoglycopeptide. It has excellent activity against Gram-positive organisms, and good penetration into skin structures, synovial fluid and bone tissue. It has a long elimination half-life of 14.4 days, which makes it a uniquely attractive agent for use in the OPAT setting. Dalbavancin is licensed for treatment of acute bacterial skin and skin structure infections with a recommended dosing regimen of 1000 mg one-off dose, and review on Day 8 for consideration of further 500 mg dose, or 1500 mg one-off dose of treatment, considered to give around 14 days effective antibiotic cover. Dalbavancin is increasingly used ‘off label’ to treat deep-seated Gram-positive infections, such as infective endocarditis, vascular graft infections and bone and joint infections. There are also descriptions of dalbavancin use as chronic suppressive therapy. Here we present three cases of longer term dalbavancin use in our OPAT service.

**Case 1:**

A 78-year-old male patient with an aortic graft infection, with no surgical option for source control. Blood culture samples grew *Staphylococcus epidermidis* and *Escherichia coli* 16 months ago. He had previously failed long-term oral suppression with co-trimoxazole alone, being readmitted with breakthrough infection. Dalbavancin alongside co-trimoxazole (for Gram-negative cover) was administered at a dose of 1500 mg Day 0, with a further 1500 mg Day 8 repeated every 2 months as a palliative management strategy. Treatment has continued for a year so far with no breakthrough infection, though the patient has had a readmission with diverticulitis.

**Case 2:**

A 58-year-old male patient with prosthetic valve enterococcal endocarditis. This followed an elective aortic root replacement with mechanical valve conduit 4 years ago. Following initial inpatient therapy, he was given long-term suppression with oral amoxicillin, but developed *Clostridioides difficile* with persistent diarrhoea. The patient was switched to dalbavancin to give a period of oral therapy to aid *C. difficile* recovery. He had three courses of dalbavancin (regimen as above) allowing improvement in his diarrhoea before being rechallenged with oral amoxicillin, which he is currently tolerating.

**Case 3:**

A 73-year-old male patient with a chronic infected right knee replacement. No recent positive microbiology, but Group G haemolytic *Strep*. was isolated 5 years ago from a knee joint sinus swab. Following review in the bone and joint clinic, it was recommended that this patient may benefit from further knee surgery, but he declined further surgical management. With no other oral options available, he has received long term suppression with dalbavancin (alternative dosing of 1000 mg every 4 weeks) and is responding well.

**Conclusions:**

Dalbavancin is increasingly used outside its licence for treatment of deep-seated infections. Here we describe the successful use of longer-term dalbavancin through OPAT. The dalbavancin was well tolerated and no side effects were reported in our cohort to date. There were no hospital re-admissions related to the original infection. The optimal dosing strategy for long-term use of dalbavancin remains unclear and requires further research. Therapeutic drug monitoring would be a helpful addition to help determine dosing but is not currently available for routine practice in the UK.

